# Visible yet Invisible: embodied meanings of weight stigma among women with binge eating disorder and higher weight

**DOI:** 10.1080/17482631.2025.2603089

**Published:** 2025-12-16

**Authors:** Kjersti Hognes Berg, Eli Natvik, Lisbeth Jahren, Trine Tetlie Eik-Nes

**Affiliations:** a Department of Neuromedicine and Movement Science, Faculty of Medicine and Health Sciences, Norwegian University of Science and Technology, Trondheim, Norway; b Stjørdal Community Mental Health Centre, Nord-Trøndelag Hospital Trust, Levanger, Norway; c Department of Health and Caring Sciences, Faculty of Health and Social Sciences, Western Norway University of Applied Sciences, Førde, Norway; d Department of Research and Innovation, Førde Hospital Trust, Førde, Norway; e Library Section for Research Support, Data and Analysis, NTNU University Library, Norwegian University of Science and Technology, Trondheim, Norway

**Keywords:** Binge eating disorder, higher-weight body, embodiment, weight stigma, shame, phenomenology

## Abstract

**Purpose:**

How the body is experienced in the context of weight stigma is largely unexplored among women with binge eating disorder (BED) and higher weight. The aim of this study was to investigate how weight stigma can manifest as embodied meaning.

**Methods:**

Eight patients participated in qualitative interviews. The data analysis was inspired by Van Manen’s hermeneutic phenomenological method. Habits, understood as unspoken patterns of behavior shaped by social and material contexts, was a central analytic concept.

**Results:**

The overarching finding was participants feeling *“Visible yet Invisible”*. Three subthemes emerged. *“Absorbed by unfitting body-size”*: experiences of a constant collision with the thin-body ideal made participants feel overtly visible and shameful. ***“**Continuous fluctuation between hyper and hypo awareness of the body”*: participants distracted themselves from overwhelming sensations attributed to body size. *“Agency based on adjustment and self-restriction”:* participants arrested their spontaneous self-expression in daily life. Habitual restrictions of bodily awareness and agency were interpreted to assist participants perceiving themselves less visible, and thus buffer feelings of shame about their body size.

**Conclusions:**

Findings highlight how weight stigma can intertwine with shame preventing habits among patients with BED and higher weight. Future directions for research on BED through patients’ embodiment are suggested.

## Introduction

Binge eating disorder (BED) is the most prevalent eating disorder (ED) (Santomauro et al., 2021), characterised by recurrent episodes of eating large amounts of food in a short period of time while experiencing loss of control, feeling distressed, and without undertaking weight compensatory behaviours (American Psychiatric Association, 2013). Although BED can appear across a broad range of body types, it is strongly associated with higher weight (Agüera et al., 2020).

Experiences of weight stigma (devaluation and social exclusion of persons with higher weight) due to weight bias (negative attitudes toward persons with higher weight) have been investigated in BED populations, indicating that weight stigma has a unique influence on psychological maladjustment, eating pathology and physiological stress (Puhl & Suh, 2015; Romano et al., 2021; Salvia et al., 2023; Wu & Berry, 2018). Weight bias works within a cultural framing that emphasises both thin-body ideals and individualised responsibility for weight control through lifestyle choices (Crossley, 2004), negatively impacting many individuals’ self-perception and health. Associations between internalised weight stigma (the process of adopting weight bias to beliefs about oneself) and a range of mental health outcomes (such as depression, anxiety, poor self-esteem and body image, disordered eating and impaired mental health related quality of life) have been identified (Pearl & Puhl, 2018). Furthermore, correlations between internalised weight bias and poor mental health seem to be higher compared to correlations between experiencing weight stigma and reduced mental health (Alimoradi et al., 2020; Emmer et al., 2020). Weight bias internalisation is however not limited to people with higher weight bodies, as a variety of eating disorder symptoms (such as binge eating, purging, restriction, excessive exercise, and muscle building) are theorised to develop in response to a common path initiated by experienced weigh stigma, through weight bias internalisation, leading to body dissatisfaction (Romano et al., 2021). Further investigation of weight bias internalisation in the context of eating disorders (EDs) is needed (Romano et al., 2021), including a better conceptualisation and operationalization of the weight bias internalisation construct (Romano et al., 2022).

Lived experiences with weight stigma as mediated through the body has been suggested as a possible pathway to provide better understandings of the weight bias internalisation process (Williams & Annandale, 2019). There are indications that weight stigma can confuse how persons with higher weight experience their bodies (Williams & Annandale, 2020), but further investigation is needed. An embodied perspective on experiences with weight stigma might expand the theorising about BED psychopathology and support better prevention and care. We define embodiment in line with Williams and Annadale as *“the fusion of the mind and body in a process whereby the society and culture within which we live are experienced in bodily terms and internalised by us: they are embodied”* (Williams & Annandale, 2019).

To obtain an overview of the empirical research literature on embodiment among people with BED and higher weight, we conducted a comprehensive literature search across six databases. A description of the search strategy can be found in the Supplementary Materials. We identified few studies exploring how this specific population experiences their bodies through perceptions and feelings. However, three studies reported that social milieus characterised by weight stigma and lack of support were central to the development of shame-driven self-perceptions and behaviour among the participants. Albertsen et al. interviewed two patients diagnosed with BED and higher weight attending a specialised unit for eating disorders about their experiences with basic body awareness therapy (Albertsen et al., 2019). Furthermore, meanings related to BED and the role of the body in BED-treatment were explored by Olsen et al. (2024) and Riise et al. (2025).

The shortage of research exploring how women with BED and higher weight experience weight stigma through their bodies in everyday-life settings motivated the present study, aiming to investigate embodied meanings of weight stigma.

### Theoretical framing

Numerous influential scholars have theorised how humans’ recurrent experiences from engaging with their surroundings can nurture the development of specific bodily modes of knowing and being (Bourdieu, 1977; Goffman, 2008; James, 1890). The French philosopher Maurice Merleau-Ponty argued that this type of knowledge about the world, and how to operate in it, works on a tacit or pre-verbal level through habits (Merleau-Ponty, 1945/2006). Furthermore he claimed that embodied knowing facilitate agency, adapted to the specific social and material context of an individual, dismissing the need of ongoing consciousness towards behavioural choices (Merleau-Ponty, 1945/2006). According to this perspective, exposure to weight stigma can shape habits that enable everyday life functioning. On the other hand, it has been emphasised that embodied adjustments to social structures of power like weight stigma can cause pain and limit spontaneous life unfoldment, especially for women (Bordo, 2020; Bruch, 1974; Dolezal, 2015; Weiss, 2013).

Building upon these theoretical insights, we aimed to investigate habits among women with BED and higher weight as intertwined with the comprehensive stigmatisation of people with higher weight in society (Shabot & Landry, 2018). In doing so, the study aligns with previous research within phenomenological psychiatry that incudes power structures, manifest in subjects’ lived bodies and self-experience, in psychopathological analysis (Zahavi & Loidolt, 2022). More specifically, the study was inspired by the works of de Beauvoir who pinpointed how notions about *man* can take on a cultural norm for *mankind*, constitutive for regarding women as “the other” (De Beauvoir, 1949/2000), and Fanon’s analysis of being black in a white-dominated world (Fanon, 1952/2008). Both de Beauvoir and Fanon argued that perceiving oneself as a deviation from a norm (“man”, “white”) can materialise in habits as a way of navigating “otherness”. Dolezal adds to this perspective by highlighting the role of chronic shame when handling feelings of inferiority (Dolezal, 2022). According to Dolezal, a recurrent social experience of feeling shameful about the body can develop into persistent and enduring affective attunement based on shame anticipation, i.e., habits to avoid feeling shame (Dolezal, 2022). Hence, the study explored how the participants’ experienced their bodies to understand whether deviations from the thin-body norm could be manifested in specific habits related to shame prevention (Guenther, 2011; Mitchell, 2020). To elaborate the analysis of how weight stigma can “get under the skin” our study was also informed by the ambiguity associated with the lived body (body-as-subject) and the physical body (body-as-object) (Merleau-Ponty, 1945/2006). Fuchs describes how these experiential poles can come into conflict among people with eating disorders (Fuchs, 2022). Due to the felt gaze of others, the body-as-object pole can accentuate through patients’ strategies to control the body in line with thin-body ideals, resulting in an alienation of the self from the body (Fuchs, 2022).

## Methods

### Recruitment and screening

In this study, we included eight women with clinical BED and higher weight. Patients were recruited from a specialised psychiatric outpatient clinic in central Norway. All patients were referred to the clinic by an obesity outpatient unit in a tertiary care hospital (implicating that their BMI was ≥40, or ≥35 if they had comorbid illness such as type 2 diabetes or heart disease), due to suspected BED. Patients underwent examination by trained clinicians in line with standard psychiatric care, and BED diagnosis was made according to diagnostic criteria using Diagnostic and Statistical Manual of Mental Disorders (DSM-5) (American Psychiatric Association, 2013). The Eating Disorder Examination-Questionnaire 6.0 (EDE-Q 6.0) (Fairburn & Beglin, 2008) was administered using the Norwegian version (Rø et al., 2010) to report a global score of eating disorder pathology. Severity of psychosocial impairment due to eating disorder was assessed using the Norwegian version (Reas et al., 2010) of the Clinical Impairment Assessment (CIA) (Bohn et al., 2008). At the time of inclusion, the Weight bias internalisation scale (Durso & Latner, 2008) was not available in Norwegian (Lussier et al., 2024). We chose to assess internalised shame, using the Internalised Shame Scale (ISS) (Cook, 2013) in Norwegian (Vikan et al., 2010), to provide a comparable measure of participants experienced levels of shame.

The recruitment period was July to December 2020. We performed purposive sampling (Gentles et al., 2015), *“attempting to gain examples of experientially rich descriptions”* (Van Manen, 2016, p. 353). Sample specificity, use of established theory, quality of dialogue, and analysis strategy have guided our assessment of information power (Malterud et al., 2016).

### Participants

The eight participants were between 25 and 54 years old, of European descent. Half of them lived alone, three lived with a partner and children, and one lived with her children only. The level of education varied from half having completed high school to the other half having a university degree. Five participants were employed full-time, two received public welfare, and one was a student. Self-reported onset of preoccupation with body and/or food was childhood or teenage for seven of the women, and one described debut in adulthood. Common characteristics were high levels of psychopathology, psychosocial impairment, and internalised shame. An overview of patients’ characteristics is displayed in Table 1.

**Table 1.** Demographic and characteristics of the participants.

Characteristics	Count
**Gender**	
Female	8
**Ethnicity**	
White	8
**Age**	
20–30	3
30–40	3
40–50	2
**Cohabiting status**	
Living alone	4
Living with partner and/or children	4
**Education level obtained**	
High school	4
University degree (minimum bachelor)	4
**Employment status**	
Full time employed	5
Student	1
Public welfare	2
**Fulfilling DSM-V criteria for binge eating disorder**	8
**Earlier treatment for other diagnosis in specialised psychiatric outpatient care**	8
**Self-reported onset of preoccupation with body and/or food**	
Pre-teen	4
Teens	3
Adulthood	1
**Eating disorder pathology**	**Mean (SD)**
EDE-Q global	4,7 (0,9)
CIA	35,5 (6,6)
Internalised shame scale	81,1 (10,1)

### Research team and reflexivity

Both the first (KHB) and fourth author (TTEN) have extensive clinical experience with specialised eating disorder treatment. (KHB) as a physiotherapist and (TTEN) as an occupational therapist. Second author (EN) is a phenomenologically oriented physiotherapist and researcher experienced in qualitative health research, mental health, and user involvement.

### Data collection

The study used a qualitative design, aiming to investigate the participants’ lived experiences. Therefore, we conducted semi-structured interviews. Six of the interviews took place at the mental health clinic, two were performed online. Each interview lasted for 60–90 minutes. Both the first and fourth author were present in each interview. (TTEN) led the conversation following a semi-structured interview guide containing themes such as everyday life function and eating disorder symptoms, experiences from previous and present clinical settings regarding weight and EDs, and post-treatment needs. The interview guide included possible follow-up questions to assist participants’ narration of their experiences. Descriptions of body awareness stem from participants dwelling on everyday life recollections. The interviews were audio-recorded and transcribed verbatim by). Acknowledging barriers to verbally representing complex lived experiences (Engelsrud & Rosberg, 2022; Van Manen, 2016), three considerations were made. First, we conducted interviews letting each participant’s unique narration lead. Second, we asked for detailed descriptions of concrete experiences rather than the participants’ attitudes, opinions, or interpretations. Third, we integrated observations of nonverbal interplay in the interview situation, that is, tone of voice, bodily movement and tension, and emotional expressions in the interview transcripts.

### Data analysis

This analysis was inspired by Van Manen’s hermeneutic phenomenological method (Van Manen, 2016). We first conducted a wholistic reading of the interviews, identifying three themes: bodily presence (experiencing the body through perceptions and emotions), bodily absence (experiencing a body-mind split: “I am not my body,” avoiding body awareness), and body-self-reflection (experiencing alienation, playing a role). This was followed by a second reading, leading to the formulation of a narrative anecdote (Van Manen, 2016) based on a condensate of one woman’s experiences resonating with the descriptions of the entire group. Feeling frustration and hopelessness having the “wrong body” now emerged as central to the participants’ bodily awareness, influencing both self-perception and management of social engagement. We proceeded with a third reading guided by “insight cultivators” (Van Manen, 2016, pp. 324-27). In this analytical step the themes were filtered through the lens of two theoretical concepts: *the body experienced as ‘the other’* (De Beauvoir, 1949/2000; Fanon, 1952/2008) and *conflict of embodiment* (Fuchs, 2022). By introducing these concepts, we sought to investigate if and how contextual (weight stigma) and ontological (being a body vs. having a body) aspects were related to participants’ descriptions of how they experienced their bodies. In our presentation of the meaning structure, “feeling visible yet invisible” represents the most abstract level of analysis, and the themes *Absorbed by unfitting body-size, Continuous fluctuation between hyper and hypo awareness of the body,* and *Agency based on adjustment and self-restriction* describe nuances*.* The latter two themes were further divided into two subthemes, depicting how feeling visible and invisible materialised in both self-perception and agency. The first author wrote draughts, and the findings were discussed among the authors at each stage of the analysis.

## Results

Feeling visible yet invisible was an overarching finding in this study investigating embodied meanings of weight stigma among women with BED and higher weight. The meaning structure was elaborated through themes and subthemes. An overview of the results is presented in Table 2.

**Table 2.** Overview of the results.

Overarching finding	Themes	Subthemes
Feeling Visible yet Invisible	Absorbed by unfitting body-size	
	Continuous fluctuation between hyper and hypo awareness of the body	The noisy body
		The muted body
	Agency based on adjustment and self-restriction	The adjusting body
		The restricted body

### Absorbed by unfitting body-size

Body size dominated the participants’ attention in social situations or in public places. Participants perceived themselves as occupying too much space, either when feeling the gaze of other people or when engaging with material artifacts such as chairs, safety belts, or public transport.

*I feel like I’m sticking out, I feel that people are watching me. It makes me feel like crying. I am so afraid people will whisper among each other “oh my god, that woman” when I enter a room.* Participant 6.

A constant eagerness to minimise or hide themselves by consciously arranging their clothes to cover their bodily curves, placing themselves in the background of a room, or not letting the body fully lean towards the ground, was described.

*Before sitting down, I sort of analyse the chair. Is it solid? Will it carry me? Or if it’s a sofa. Will I destroy the stuffing in the pillows? Will everyone see that I sat there?* Participant 7.

Furthermore, being reminded of unfitting body size paralleled feelings of shame. Having a body others would label as too big was by the participants described as *the* shame:

*Shame is being huge, thick, ugly, and that no-one likes me. I have been repeating this in my head since childhood [pauses] huge and ugly…. Ahhhhh…… I feel very dizzy… these are bad memories [lowering her voice, laughs a kind of laughter then pauses for many seconds].* Participant 7.

Recalling feeling thick, ugly, and unlikeable throughout her life seemed to trigger a specific response in the participant. As she described feeling dizzy, the tone of her voice lowered before she was quiet for many seconds. Her attention moved from talking about her body from a distance to experiencing the actual body here and now. Describing shame made it appear, making the body too present to ignore, hindering continuity in the conversation.

The women’s awareness seemed to be absorbed with their bodies’ visibility, making them perceive the body as a thing to be validated and handled. This feeling was underpinned by family and friends repetitively commenting on the participants’ body and weight during childhood and puberty, continuing into adulthood by others (partners, friends, healthcare providers), remarking their weight and shape-based looks. In addition, objects such as short safety belts and flimsy furniture would also remind them of their bodies that did not fit in. Recognising constant devaluation due to body size, the women described feelings of shame and made a lot of effort to modify their body appearance. By covering curves, exercising, and dieting, and adapting their bodily interaction with the material surroundings, the participants attempted to align with the perceived expectations of what a body should look like and accommodate. By staying large-bodied, the participants felt inferior and doomed. Unfortunately, they did not acknowledge that the body developed its wrongness in a cascade of situations that were outside the participants’ power to (re)define, and that their body constantly collided with the thin-body ideal both materially and relationally.

### Continuous fluctuation between hyper and hypo awareness of the body

The actual or imagined condescending gaze of the other appeared to frame the participants’ perceptual experience of the body as an ongoing alternation between hyper awareness and hypo awareness. The body emerged as either overly visible (noisy) or invisible (muted).

#### The noisy body

The feelings of discomfort, heaviness, fatigue, dizziness, and pain when approaching the body were described by the participants. Hence, body awareness was primarily perceived as distressing.

*I am tired of feeling exhausted all the time, of feeling the body ache and lack energy.* Participant 2.

The women also talked about their lust, relaxation, and comfort. These experiences were sporadic and related to planning or devouring a meal in binge eating episodes; however, they were always replaced by distress and feelings of shame, guilt, and hopelessness. Thus, positive bodily experiences were described as transient. Being aware of their bodies was described as intensely consuming and overwhelming, which made them want to escape.

*I don’t want to think about food, stress, all the things I do wrong. I want to hide from it all: things, concerns, thoughts, and the feelings of not being good enough.* Participant 3.

What they noticed in their bodies seemed to stem directly from perceiving the body as being too big due to others devaluating gaze or interacting with objects revealing the body’s unfittingness. Awareness of a broader range of information from the body interacting with the surroundings, such as basic physiological needs, joy, interest, or anger, allowing life unfoldment, self-care, and boundary settings, was at its best transient, but mostly deafened by the painful experiences in the participants’ descriptions.

#### The muted body

Numbness after eating until very full was described as an experience of distancing themselves from bodily perceptions; hence, binge eating appeared to be a way out of what they experienced as unbearable noise from the body and of managing distress. In addition to binge eating, external auditory stimulation had a masking effect on distressful bodily awareness:

*Silence is scary. I don’t know, but I hate being alone, managing to take a shower or drive the car or doing housework when alone, I need to listen to music or a podcast. I need noise from outside. If I just sit still, I have nowhere to escape.* Participant 2.

Furthermore, keeping themselves occupied appeared to be a third strategy to deafen disturbing feelings, dominating their bodily awareness. The women described staying busy as a deliberate strategy to keep their distress out of consciousness. Doing housework, performing repetitive movements, or scrolling on the mobile phone was experienced as a conscious practice to distance oneself from the body. In addition, muting was described as a more continuous way of experiencing the body:

*I think I just muted myself at a certain point, and I have just continued doing that. I have in a way forgotten myself, and now I don’t know where to start. What do I want? I have tried to address things, and been in therapy twice, but I haven’t managed to get hold of it.* Participant 8.

Participants experienced that the body could feel divided and that the neck represented a split separating the head from the rest of the body, making them imagine that they existed only as minds. Feeling numb, encapsulated, or disconnected were other words describing the experience of being physically detached. Participants could also explicitly state “*I am not my body*,” expressing that distancing their bodies worked to protect against feeling vulnerable. “*Putting on a mask*,” was another formulation used to describe bodily detachment as a shield against revealing perceptual and emotional distress and chaos.

The participants experienced their bodies through continuous fluctuation between noticing too much and too little. Therefore, it seemed difficult for the women to indulge in conscious dwellings on nuanced bodily perceptions, indicating physiological or emotional needs in concrete situations. Unawareness of perceptions such as hunger, exhaustion, affection, engagement, or aversion seemed to leave the women poorly equipped to experience a robust, embodied self when engaging with the world around. Further, lack of nuanced bodily information was associated with ambivalence and frustration in situations that demanded a deliberate choice, such as planning their daily schedule, deciding how to respond to invitations, family and friends appeal for practical help, etc. Being either perceptually over-noticeable or blurred to themselves appeared to push the participants’ social participation towards adjusting to others’ expectations or withdrawal through self-restriction.

### Agency based on adjustment and self-restriction

#### The adjusting body

In addition to adjusting to social expectations and demands due to confusion about their own needs and preferences, the participants described performing conscious social adjustments to reduce their visibility and hence shame. This was accomplished by arresting vitality and self-expression:

*I used to think that I don’t have the right to be angry because I am fat, I don’t have the right to… anything because I am so fat. But then I forget about all the restrictions I put on myself; I feel joy and forget about being fat. Coming home, I regret taking up space, drawing attention to myself…. Because I shall not be visible.*

*When I am alone, I always think: There is no one who likes you, you talk too loudly, you laugh too loud, you are too intense, it is too much of you. Can’t you just pull yourself together, sit quietly in your chair and be quiet? But I can’t [she cries while talking].* Participant 6.

Being diligent and attentive to other people’s needs was another approach to social adjustment:

*I think that slim people look at me and think I am a weak person. It is a way of thinking which has stuck in me. I grew up being invisible, you know—not saying anything. I don’t recall when I started thinking about being the kindest and cleverest. My siblings revolted, I didn’t. I cleaned the entire house, vacuumed, washed, took out all the carpets, mowed the lawn and shovelled snow, all before I was asked to.* Participant 7.

The same woman also described that others would disapprove of her behaviour because it was considered inappropriate for a girl:

*I opposed my aunt when she wanted me to wear a dress, but it was very uncomfortable. I felt that I was not considered good enough, that I was not girly enough. I have always been a tomboy, climbing trees, riding horses and even cows [laughs]. I have sometimes wondered if I am a boy. But I am not [laughs again].* Participant 7.

Adjusting to approved behaviour, such as being clever, quiet, and attentive, seemed to be a strategy to make their bodies less visible and thus feel more accepted by others. Opposing or freeing themselves from this very restricted life unfoldment seemed disturbing or somewhat unreachable. Instead, they described ways to ignore the impulses of resistance and perform assertiveness.

#### The restricted body

Simultaneous with the feeling of being overly visible due to their body size, the participants felt invisible, as humans having a multitude of other characteristics. They experienced paradoxical visible invisibility due to feelings of being reduced to shape and weight, of not being acknowledged as persons with a variety of talents, knowledge, and agency. Moreover, the lack of feeling seen as a whole person could manifest as self-restriction. One woman described:

*I made a list, as long as my height, with all the names and comments others had given me because of my body size. I called it my motivation-list for weight reduction. A year ago, I decided to burn the list together with friends with similar experiences. We burned the lists because they witnessed how we limit ourselves.* Participant 4.

Feeling recognised merely as bigger bodied and not as a whole multi-faceted person capable of accomplishing things was accompanied by despair, hopelessness, inadequacy, sorrow, anger, loss of control, shame, and self-contempt. Overeating was experienced by the participants as a strategy to numb or comfort these painful feelings, but was also described as a method to arrest impulses of engagement and ambition:

*When I eat, I numb the value of myself…. I don’t allow myself to succeed …. Not being kind to myself is sort of a coping strategy. When I was 19, I auditioned for a school of performing arts. I made it through selection after selection, but before the final test I went home without taking the chance.* Participant 6.

Thus, handling body size visibility by adjusting (blending in, making themselves acceptable) or holding back engagement (avoiding making themselves more visible) possibly reinforced notions of invisibility.

Taken together, the women’s bodily engagement with the world seemed disturbed. Feeling visible yet invisible both to themselves and others due to higher weight appeared to weaken their sense of being an embodied self, directing their life unfoldment towards adjustment to others’ expectations rather than letting their own preferences decide.

## Discussion

The present study adds to the scarce literature examining BED psychopathology through patients’ embodied experiences and highlights how weight stigma can interact with patients' habits in everyday life. The participants experienced that their bodies demanded explicit attention because they frequently collided with thin-body ideals, making them feel inferior to others. Feeling shameful about their body-size visibility appeared to fuel habits making the body less visible to themselves and others, such as restricting general bodily awareness and inhibiting participation and self-fulfilment. Our findings are consistent with explorative research on other types of EDs reporting that patients experienced the body as overly visible, an obstacle for self-recognition, others’ recognition, and interfered with their actions (Mancini et al., 2021). Furthermore, participants’ preoccupation with their body size and consecutive development of ED-behaviour are in line with empirical research regarding the role of body image (i.e., overvaluation of weight and shape for self-worth) in BED pathology (Grilo et al., 2024, 2013; Lewer et al., 2017; Wang et al., 2019). Moreover, descriptions of shame-driven behaviour by the participants in our study correspond with a meta-analysis identifying strong associations between body shame and psychopathology in ED subjects generally (Nechita et al., 2021), and BED subjects specifically (O'Loghlen et al., 2022). Exposure to thin-body ideals, explicit attention to their bodies, and feeling shameful may have disrupted the body’s function as a silent prerequisite for life unfoldment associated with health (Merleau-Ponty, 1945/2006; Slatman, 2022). These findings will now be discussed in more detail.

### Chronic body-shame

The participants described that their bodies had been negatively commented on by others from an early age, including primary caregivers, making them feel shameful and inferior to others. In the literature, shame has been defined as a self-conscious emotion, typically triggered in situations of failing to live up to social values (such as the thin-body norm) (Bruch, 1974; Goss & Allan, 2009). Participants’ experiences of feeling devaluated in their present lives appeared to stem from an inherent expectation of feeling shame in social situations. It was as if “old shame” made them expect feeling shameful “here-and-now”. This finding is supported by a study investigating shame experiences from childhood and adolescence among women with BED, reporting that *“The traumatic and centrality qualities of shame-related memories predicted current external shame, especially body image shame”* (Duarte et al., 2017, p. 511). Thus, it can be argued that the participants encounters with shame in their formative years might have contributed to what Dolezal described as shame-driven habits or chronic shame: *“avoiding shame becomes a central feature of subjectivity, and an absent, but anticipated, shame becomes the negative space around which experience is organised” (Dolezal, 2022*, p. *746)*. In the following paragraph we discuss the patients’ descriptions of limited self-awareness and agency with the concept of chronic shame as a backdrop, e.g., as habits to avoid feeling shameful among others due to not living up to societal body-norms.

### Habits as shame prevention

Participants perceived their bodies overwhelmed or detached through a continuous shift between hyper awareness of their body size and hypo awareness of perceptions and feelings. Fluctuation of bodily modes has been thoroughly described in the literature on trauma (Levine, 1997; Ogden et al., 2006; Van der Kolk, 2014), and was found in studies investigating bodily experiences among patients with BED and higher weight. Albertsen and co-workers reported participants “*feeling fragmented, trying to avoid perceived unpleasant aspects about their body weight and shape, difficult emotions, own history, and pain, all rooted in the body”* (Albertsen et al., 2019 p. 9). Furthermore, in a similar study, participants' experiences were dominated either by the body’s size (feeling heavy, occupying space) or by absence: *“Detachment from body to avoid contact with emotions and being visible”* (Olsen et al., 2024 p. 6).

In our study, participants appeared to experience their bodies either through perceptions, thoughts, and feelings related to body weight or shape or through absence, resonates with what Fuchs described as conflict of embodiment, where the *body as object* dominates the *body as subject* (Fuchs, 2022). Being absorbed with weight, shape, and visibility (body as object), patients may suppress undesirable perceptions and feelings such as shame (body as subject) (Gaete & Fuchs, 2016). However, awareness of perceptions and feelings is in our theoretical framing central to embodied subjectivity, as it is the basic source of meaningful orientation and decision-making (e.g., boundary setting and self-care related to hunger, satiety etc.) (Fuchs, 2022; Gaete & Fuchs, 2016). According to Fuchs *“[The] subject body conveys a vague interoceptive background feeling, for example of wellbeing or discomfort, of vitality, energy, or fatigue; it is also the source of drives, impulses and desires which the subject experiences either pre-reflectively or explicitly”* (Fuchs, 2022 p. 110)*.* Hence, by restricting bodily awareness people with eating disorders may experience alienation of the self from the body (Fuchs, 2022). In our study this may explain the patients’ descriptions of a felt division between mind and body, and their experience of ambivalence and frustration when navigating their everyday lives. Distancing themselves from their body appeared to buffer against feeling shameful about their body size but simultaneously, it constrained their awareness of perceptual information about physiological and emotional needs.

Furthermore, engagement with the surroundings was experienced by participants as disrupted due to either adjusting to other people’s expectations or by withholding spontaneous involvement. Our finding contrasts with Albertsen and co-workers who reported everyday life to be less affected by patients’ fluctuating bodily awareness: *“they were still able to perform their usual activities such as taking care of their children and participating in working life. The experienced split was related only to some aspects of their own body and the everyday world”* (Albertsen et al., 2019 p. 10). Olsen and colleagues on the other hand, found that participants with BED and higher weight experienced bodily detachment as necessary to cope with everyday activities (Olsen et al., 2024). Likewise, Riise et al. found that adjustments due to *"body shame led to withdrawal from activities and social isolation"* among patients with BED (Riise et al., 2025 p. 7). Concurrent with our findings, limitations of life unfoldment have been described in qualitative research on all types of EDs (Mancini et al., 2021). Shame-based adjustment through restricted agency and participation, has also been explored in the context of sexual or gender-based oppression (Ahmed, 2020; Young, 1980), illustrated by the following passage: *“One cannot confidently use ones’ body in strenuous physical settings or work deliberately toward complex projects that demand one’s full attention if one cannot feel comfortable without monitoring one’s appearance”* (Welsh, 2022 p. 67).

Seen through the lens of chronic shame the participants’ tendency to fluctuate between hyper and hypo awareness of the body, adjust to other people’s expectations or withhold spontaneous involvement, can be interpreted as meaningful shame preventing attunements (Dolezal, 2022). Thus, our findings may indicate that weight stigma can interfere and influence habits among patients with BED and higher weight. To handle shame caused by the higher weight body’s visibility, body awareness and agency can be structured around making the body invisible and thus diminish the harmful effects of stigma. In other words, this study may illustrate how weight stigma can be internalized through habits.

### Methodological considerations and future research

Applying a phenomenological methodology has brought the situation of the participants at the forefront of the analysis, illuminating their experiences as meaningful given specific contextual factors. Thus, the methodology facilitated exposure of weight stigma, conveyed in relations and material surroundings, as a central condition for experiencing disturbed self-awareness and agency in participants with BED and higher weight. Recruiting participants from an outpatient treatment context, with BED diagnosis confirmed by specialists using diagnostic manuals, contributes to the transferability of findings to other clinical settings. Furthermore, the first and fourth authors’ familiarity with the patient population could be considered a strength of this study as it supported the creation of a rich and nuanced material. The first author’s clinical experience with Norwegian Psychomotor Physiotherapy can be regarded as an epistemological advantage when analysing the material (Haraway, 2016; Harding, 2001), as the body is understood as a basic site for knowledge and meaning-making in this method (Ekerholt & Bergland, 2022).

The study also has limitations. Researching embodied meanings of a phenomenon, such as weight stigma, is associated with methodological challenges (Brown et al., 2011). As interviews rely on participants’ verbal expressions, they can be insufficient for grasping the complexity of embodied experience (Van Manen, 2016 p. 314). This point calls for innovative approaches, and future research should consider methods allowing participants’ implicit or pre-verbal experiences to unfold more fully than interviews do (Murray et al., 2023). Moreover, the findings represent a single study in an understudied field. Hence, further investigation is needed to elaborate understandings of how weight stigma might relate to BED-psychopathology through patients’ embodiment. Associations with sensations like hunger and pain, binge eating, and the possible impact of childhood trauma (like neglect and sexual abuse), are relevant aspects for further exploration. Future investigations should also study how sexual orientation, colour, and socioeconomic status might interfere with weight stigma, feelings of shame, and habits among patients with BED and higher weight. Furthermore, as the habits described in this study operate on a tacit or pre-verbal level, researching body oriented or movement-based interventions tailored to facilitate nuanced bodily awareness as basis for agency could be beneficial in future studies.

## Data Availability

To ensure the full anonymity of the participants, the data generated and analysed during the current study are not publicly available. The transcribed interviews are available from the corresponding author upon reasonable request.

## References

[cit0001] Agüera, Z., Lozano-Madrid, M., Mallorquí-Bagué, N., Jiménez-Murcia, S., Menchón, J. M., & Fernández-Aranda, F. (2020). A review of binge eating disorder and obesity. *Neuropsychiatrie: Klinik, Diagnostik, Therapie und Rehabilitation: Organ der Gesellschaft Osterreichischer Nervenarzte und Psychiater*, *35*(2), 57–67. 32346850 10.1007/s40211-020-00346-w

[cit0002] Ahmed, S. (2020). *Queer phenomenology: Orientations, objects, others*. Duke University Press.

[cit0003] Albertsen, M. N., Natvik, E., & Råheim, M. (2019). Patients’ experiences from basic body awareness therapy in the treatment of binge eating disorder-movement toward health: A phenomenological study. *Journal of Eating Disorders*, *7*(1), 1–12. 10.1186/s40337-019-0264-0 31641506 PMC6802330

[cit0004] Alimoradi, Z., Golboni, F., Griffiths, M. D., Broström, A., Lin, C. Y., & Pakpour, A. H. (2020). Weight-related stigma and psychological distress: A systematic review and meta-analysis. *Clinical Nutrition*, *39*(7), 2001–2013. 10.1016/j.clnu.2019.10.016 31732288

[cit0005] American Psychiatric Association, D. (2013). *Diagnostic and statistical manual of mental disorders: DSM-5*. Washington, DC: American psychiatric association.

[cit0006] Bohn, K., Doll, H. A., Cooper, Z., O'Connor, M., Palmer, R. L., & Fairburn, C. G. (2008). The measurement of impairment due to eating disorder psychopathology. *Behaviour Research and Therapy*, *46*(10), 1105–1110. 10.1016/j.brat.2008.06.012 18710699 PMC2764385

[cit0007] Bordo, S. R. (2020). The body and the reproduction of femininity: A feminist appropriation of Foucault. In *The New Social Theory Reader* (pp. 207–218). Routledge. 10.4324/9781003060963-33

[cit0008] Bourdieu, P. (1977). *Outline of a theory of practice*. Cambridge University Press.

[cit0009] Brown, S. D., Cromby, J., Harper, D. J., Johnson, K., & Reavey, P. (2011). Researching “experience”: Embodiment, methodology, process. *Theory & Psychology*, *21*(4), 493–515. 10.1177/0959354310377543

[cit0010] Bruch, H. (1974). *Eating disorders. Obesity, anorexia nervosa, and the person within.* Routledge & Kegan Paul.

[cit0011] Cook, D. R. (2013). Measuring shame: The internalized shame scale. In *The treatment of shame and guilt in alcoholism counseling* (pp. 197–215). Routledge.

[cit0012] Crossley, N. (2004). Fat is a sociological issue: Obesity rates in late modern,‘body-conscious’ societies. *Social Theory & Health*, *2*(3), 222–253. 10.1057/palgrave.sth.8700030

[cit0013] De Beauvoir, S. (1949/2000). Le deuxième sexe [Det annet kjønn] (B. Christensen, Trans.). Pax.

[cit0014] Dolezal, L. (2015). *The body and shame: Phenomenology, feminism, and the socially shaped body*. Lexington Books.

[cit0015] Dolezal, L. (2022). The horizons of chronic shame. *Human Studies*, *45*(4), 739–759. 10.1007/s10746-022-09645-3 36483088 PMC7613895

[cit0016] Duarte, C., Pinto-Gouveia, J., & Ferreira, C. (2017). Ashamed and fused with body image and eating: Binge eating as an avoidance strategy. *Clinical Psychology & Psychotherapy*, *24*(1), 195–202. 10.1002/cpp.1996 26689603

[cit0017] Durso, L. E., & Latner, J. D. (2008). Understanding self-directed stigma: Development of the weight bias internalization scale. Obesity (Silver Spring, Md.), *16 Suppl 2*, S80–86. 10.1038/oby.2008.448 18978768

[cit0018] Ekerholt, K., & Bergland, A. (2022). Embodied knowledge–the phenomenon of subjective health complaints reflected upon by norwegian psychomotor physiotherapy specialists. *Physiotherapy Theory and Practice*, 38(12), 2122–2133. 33957856 10.1080/09593985.2021.1920073

[cit0019] Emmer, C., Bosnjak, M., & Mata, J. (2020). The association between weight stigma and mental health: A meta-analysis. *Obesity reviews*, *21*(1), e12935. 10.1111/obr.12935 31507062

[cit0020] Engelsrud, G., & Rosberg, S. (2022). Theorizing bodily dialogs–reflection on knowledge production in phenomenological research. *Physiotherapy Theory and Practice*, *38*(12), 1833–1842. 10.1080/09593985.2021.1923098 33974495

[cit0021] Fairburn, C. G., & Beglin, S. J. (2008). Eating disorder examination questionnaire. In C. G. Fairburn (Ed.), *Cognitive Behavior Therapy and Eating Disorders* (pp. 309–313). Guilford Press.

[cit0022] Fanon, F. (1952/2008). Peau Noire, Masques Blancs [Black Skin, White Masks] (R. Philcox, Trans.). Penguin Books.

[cit0023] Fuchs, T. (2022). The disappearing body: Anorexia as a conflict of embodiment. Eating and Weight Disorders, *27*(1), 109–117. 10.1007/s40519-021-01122-7 33666885 PMC8860785

[cit0024] Gaete, M. I., & Fuchs, T. (2016). From body image to emotional bodily experience in eating disorders. *Journal of Phenomenological Psychology*, *47*(1), 17–40. 10.1163/15691624-12341303

[cit0025] Gentles, S. J., Charles, C., Ploeg, J., & McKibbon, K. A. (2015). Sampling in qualitative research: Insights from an overview of the methods literature. *The Qualitative Report*, *20*(11), 1772–1789.

[cit0026] Goffman, E. (2008). *Behavior in public places*. Simon and Schuster.

[cit0027] Goss, K., & Allan, S. (2009). Shame, pride and eating disorders. *Clinical Psychology & Psychotherapy: An International Journal of Theory & Practice*, *16*(4), 303–316. 10.1002/cpp.627 19639646

[cit0028] Grilo, C. M. (2013). Why no cognitive body image feature such as overvaluation of shape/weight in the binge eating disorder diagnosis? The International Journal of Eating Disorders, *46*(3), 208–211. 10.1002/eat.22082 23233198 PMC3600067

[cit0029] Grilo, C. M., Ivezaj, V., & Gueorguieva, R. (2024). Overvaluation of shape/weight at posttreatment predicts relapse at 12-month follow-up after successful behaviorally-based treatment of binge-eating disorder. *International Journal of Eating Disorders*, *57*, 1268–1273. 10.1002/eat.24141 38321617 PMC11093697

[cit0030] Guenther, L. (2011). Shame and the temporality of social life. *Continental Philosophy Review*, *44*, 23–39. 10.1007/s11007-011-9164-y

[cit0031] Haraway, D. (2016). ‘Situated Knowledges: The science question in feminism and the privilege of partial perspective'. In *Space, gender, knowledge: Feminist readings* (pp. 53–72). Routledge.

[cit0032] Harding, S. (2001). Feminist standpoint epistemology. In M. Lederman, & I. Bartsch (Eds.), *The Gender and Science Reader* (pp. 145–168). London and New York: Routledge.

[cit0033] James, W. (1890). Habit. H. Holt.

[cit0034] Levine, P. A. (1997). *Waking the tiger: Healing trauma: The innate capacity to transform overwhelming experiences*. North Atlantic Books.

[cit0035] Lewer, M., Bauer, A., Hartmann, A. S., & Vocks, S. (2017). Different facets of body image disturbance in binge eating disorder: A review. Nutrients, *9*(12), 1294. 10.3390/nu9121294 29182531 PMC5748745

[cit0036] Lussier, T., Tangen, J. H. Q., Eik-Nes, T. T., Karlsen, H. R., Berg, K. H., & Fiskum, C. (2024). Testing the validity of the Norwegian translation of the modified weight bias internalization scale. *Journal of Eating Disorders*, *12*(1), 117. 10.1186/s40337-024-01067-z 39148088 PMC11325566

[cit0037] Malterud, K., Siersma, V. D., & Guassora, A. D. (2016). Sample size in qualitative interview studies: Guided by information power. *Qualitative Health Research*, *26*(13), 1753–1760. 10.1177/1049732315617444 26613970

[cit0038] Mancini, M., Mignogna, S., & Stanghellini, G. (2021). Dear body… An explorative study on anomalous bodily experiences in persons with feeding and eating disorders. Psychopathology (Basel), *54*(5), 242–252. 10.1159/000517505 34350886

[cit0039] Merleau-Ponty, M. (1945/2006). Phénomènologie de la perception [Phenomenology of perception] (C. Smith, Trans.). Routledge.

[cit0040] Mitchell, D. (2020). Sartre and Fanon: The phenomenological problem of shame and the experience of race. *Journal of the British Society for Phenomenology*, *51*(4), 352–365. 10.1080/00071773.2020.1732577

[cit0041] Murray, A., Steffen, M., Keiller, E., Turri, M. G., & Lau, J. Y. (2023). Body mapping for arts-based inquiry in mental health research: A scoping review. *The Lancet Psychiatry*, *10*(11), 896–908. 10.1016/S2215-0366(23)00224-9 37611618

[cit0042] Nechita, D. M., Bud, S., & David, D. (2021). Shame and eating disorders symptoms: A meta-analysis. *International Journal of Eating Disorders*, *54*(11), 1899–1945. 10.1002/eat.23583 34302369

[cit0043] Ogden, P., Minton, K., & Pain, C. (2006). *Trauma and the body: A sensorimotor approach to psychotherapy (norton series on interpersonal neurobiology)*. WW Norton & Company.

[cit0044] O'Loghlen, E., Grant, S., & Galligan, R. (2022). Shame and binge eating pathology: A systematic review. *Clinical Psychology & Psychotherapy*, *29*(1), 147–163. 10.1002/cpp.2615 34010473

[cit0045] Olsen, H. T., Vangen, S. B., Stänicke, L. I., & Vrabel, K. (2024). “I feel so small and big at the same time”—exploring body experience and binge eating disorder following inpatient treatment: A qualitative study. *Frontiers in Psychology*, *15*, 1432011. 10.3389/fpsyg.2024.1432011 39469244 PMC11513874

[cit0046] Pearl, R. L., & Puhl, R. M. (2018). Weight bias internalization and health: A systematic review. *Obesity Reviews*, *19*(8), 1141–1163. 10.1111/obr.12701 29788533 PMC6103811

[cit0047] Puhl, R., & Suh, Y. (2015). Stigma and eating and weight disorders. *Current Psychiatry Reports*, *17*(3), 1–10. 10.1007/s11920-015-0552-6 25652251

[cit0048] Reas, D. L., Rø, Ø., Kapstad, H., & Lask, B. (2010). Psychometric properties of the clinical impairment assessment: Norms for young adult women. *International Journal of Eating Disorders*, *43*(1), 72–76. 10.1002/eat.20653 19260038

[cit0049] Riise, J., Gulliksen, K. S., Vrabel, K., & Halvorsen, M. S. (2025). “Binge eating disorder is the slum of eating disorders”: a qualitative study of Norwegian women with binge eating disorder in the encounter with the healthcare system. Journal of Eating Disorders, 13(1), 1–14. 10.1186/s40337-025-01223-z 40108713 PMC11921570

[cit0050] Rø, Ø., Reas, D. L., & Lask, B. (2010). Norms for the eating disorder examination questionnaire among female university students in Norway. *Nordic Journal of Psychiatry*, *64*(6), 428–432. 10.3109/08039481003797235 20429744

[cit0051] Romano, K. A., Heron, K. E., & Henson, J. M. (2021). Examining associations among weight stigma, weight bias internalization, body dissatisfaction, and eating disorder symptoms: Does weight status matter? *Body Image*, *37*, 38–49. 10.1016/j.bodyim.2021.01.006 33556915 PMC8187267

[cit0052] Romano, K. A., Heron, K. E., Sandoval, C. M., Howard, L. M., MacIntyre, R. I., & Mason, T. B. (2022). A meta-analysis of associations between weight bias internalization and conceptually-related correlates: A step towards improving construct validity. *Clinical Psychology Review*, *92*, 102127. 10.1016/j.cpr.2022.102127 35074712 PMC8858873

[cit0053] Salvia, M. G., Ritholz, M. D., Craigen, K. L., & Quatromoni, P. A. (2023). Women's perceptions of weight stigma and experiences of weight-neutral treatment for binge eating disorder: A qualitative study. *EClinicalMedicine*, *56*, 101811. 10.1016/j.eclinm.2022.101811 36618893 PMC9816903

[cit0054] Santomauro, D. F., Melen, S., Mitchison, D., Vos, T., Whiteford, H., & Ferrari, A. J. (2021). The hidden burden of eating disorders: An extension of estimates from the global burden of disease study 2019. *The Lancet Psychiatry*, *8*(4), 320–328. 10.1016/S2215-0366(21)00040-7 33675688 PMC7973414

[cit0055] Shabot, S. C., & Landry, C. (2018). The water we swim in: Why feminist phenomenology today, *Rethinking Feminist Phenomenology: Theoretical and Applied Perspectives* (Vol. *1*, pp. 1–10). 10.5040/9798881812256.0004

[cit0056] Slatman, J. (2022). Toward a phenomenology of abnormality. In S. B. a. T. Welsh (Ed.), *Normality, Abnormality, and Pathology in Merleau-Ponty* (pp. 19–39). Suny Press. 10.1515/9781438486871-003

[cit0057] Van der Kolk, B. (2014). *The body keeps the score: Brain, mind, and body in the healing of trauma*. Penguin Books.

[cit0058] Van Manen, M. (2016). *Phenomenology of practice: Meaning-giving methods in phenomenological research and writing*. Routledge.

[cit0059] Vikan, A., Hassel, A. M., Rugset, A., Johansen, H. E., & Moen, T. (2010). A test of shame in outpatients with emotional disorder. *Nordic Journal of Psychiatry*, *64*(3), 196–202. 10.3109/08039480903398177 19919290

[cit0060] Wang, S. B., Jones, P. J., Dreier, M., Elliott, H., & Grilo, C. M. (2019). Core psychopathology of treatment-seeking patients with binge-eating disorder: A network analysis investigation. *Psychological Medicine*, *49*(11), 1923–1928. 10.1017/S0033291718002702 30232948 PMC7194445

[cit0061] Weiss, G. (2013). *Body images: Embodiment as intercorporeality*. Routledge.

[cit0062] Welsh, T. (2022). *Feminist existentialism, biopolitics, and critical phenomenology in a time of bad health*. Taylor & Francis.

[cit0063] Williams, O., & Annandale, E. (2019). Weight bias internalization as an embodied process: Understanding how obesity stigma gets under the skin. Frontiers in Psychology, *10*, 953. 10.3389/fpsyg.2019.00953 31110487 PMC6501757

[cit0064] Williams, O., & Annandale, E. (2020). Obesity, stigma and reflexive embodiment: Feeling the ‘weight’of expectation. *Health*, *24*(4), 421–441. 10.1177/1363459318812007 30428717

[cit0065] Wu, Y. K., & Berry, D. C. (2018). Impact of weight stigma on physiological and psychological health outcomes for overweight and obese adults: A systematic review. *Journal of Advanced Nursing*, *74*(5), 1030–1042. 10.1111/jan.13511 29171076

[cit0066] Young, I. M. (1980). Throwing like a girl: A phenomenology of feminine body comportment motility and spatiality. *Human Studies*, *3*(1), 137–156. 10.1007/BF02331805

[cit0067] Zahavi, D., & Loidolt, S. (2022). Critical phenomenology and psychiatry. *Continental Philosophy Review*, *55*(1), 55–75. 10.1007/s11007-021-09553-w

